# Antagonistic Potential of Novel Endophytic *Bacillus* Strains and Mediation of Plant Defense against Verticillium Wilt in Upland Cotton

**DOI:** 10.3390/plants9111438

**Published:** 2020-10-25

**Authors:** Nadeem Hasan, Ayaz Farzand, Zhou Heng, Irfan Ullah Khan, Anam Moosa, Muhammad Zubair, Yang Na, Sun Ying, Tang Canming

**Affiliations:** 1 State Key Laboratory of Crop Genetics and Germplasm Enhancement, College of Agriculture, Nanjing Agricultural University, Nanjing 210095, China; 2017201097@njau.edu.cn (N.H.); 2019201046@njau.edu.cn (Z.H.); 2017201095@njau.edu.cn (I.U.K.); 2018101098@njau.edu.cn (Y.N.); 2016101118@njau.edu.cn (S.Y.); 2 Department of Plant Pathology, University of Agriculture, Faisalabad 38040, Pakistan; ayaz.farzand@uaf.edu.pk (A.F.); annuanum24@gmail.com (A.M.); 2017202058@njau.edu.cn (M.Z.)

**Keywords:** *Verticillium dahliae*, cotton, *B. altitudinis*, *B. velezensis*, lipopeptides, defense-related genes

## Abstract

Verticillium wilt caused by *Verticillium dahliae* is a threatening disease of cotton, causing economic loss worldwide. In this study, nine endophytic *Bacillus* strains isolated from cotton roots exhibited inhibitory activity against *V. dahliae* strain VD-080 in a dual culture assay. *B. altitudinis* HNH7 and *B. velezensis* HNH9 were chosen for further experiments based on their high antagonistic activity. The secondary metabolites of HNH7 and HNH9 also inhibited the growth of VD-080. Genetic marker-assisted detection revealed the presence of bacillibactin, surfactin, bacillomycin and fengycin encoding genes in the genome of HNH7 and HNH9 and their corresponding gene products were validated through LC-MS. Scanning electron microscopy revealed mycelial disintegration, curling and shrinkage of VD-080 hyphae after treatment with methanolic extracts of the isolated endophytes. Furthermore, a significant reduction in verticillium wilt severity was noticed in cotton plants treated with HNH7 and HNH9 as compared to control treatments. Moreover, the expression of defense-linked genes, viz., *MPK3*, *GST*, *SOD*, *PAL*, *PPO* and *HMGR*, was considerably higher in plants treated with endophytic *Bacillus* strains and inoculated with VD-080 as compared to control.

## 1. Introduction

Cotton is an important cash crop worldwide, but the crop is vulnerable to the attack of *Verticillium dahliae*, affecting the quality and quantity of the produce. *V. dahliae* associated with verticillium wilt is considered as the most destructive pathogen of cotton, causing a loss of 2.5 million hectares annually in China [1,2]. Although a few tolerant cotton varieties are available, they do not possess sufficient ability to protect the crop from economic damage. Some chemicals, such as fungicide benomyl and the plant defense activator acibenzolar-S-methyl, seem to be effective but they are not environmentally friendly [3]. The biological control using beneficial bacteria is a safer and better approach to protect the crop from economic yield loss.

The rhizosphere contains a variety of microbial communities which can protect plants through the suppression of soil-borne pathogens. Among these microbes, endophytic bacteria reside inside the plant and colonize an ecological niche like phytopathogens and can effectively be used to suppress the attack of various phytopathogens. Therefore, these endophytic bacteria are strong candidates for the biological control of many plant diseases [4,5,6,7,8,9]. *Bacillus* species such as *B. amyloliquefaciens*, *B. velezensis, B. subtilis* and *B. altitudinis* have been widely employed for their antagonistic effects against foliar, soil-borne and post-harvest fungal diseases [5,10,11,12].

*Bacillus* species can produce several antimicrobial compounds [13], such as lipopeptides (fengycin, surfactin, bacillomycin D and iturin) [14,15,16], siderophores (bacillibactin) and polyketides (bacillaene) [17]. The identification of these secondary metabolites and their corresponding antimicrobial activity is necessary to determine the biological control activity of *Bacillus* species. DNA-based genetic markers have been used to detect the presence of antimicrobial compound encoding genes that can be specifically detected through PCR amplifications [15]. Activation of induced systemic resistance (ISR) is associated with defense-linked gene expression and plants respond more rapidly against pathogen attack [9]. ISR induction triggers transcriptional regulation of plant defense genes in crop plants that in turn prevents the proliferation of plant pathogens [18,19]. Many members of *Bacillus* spp. act as plant safeguards against phytopathogens through the induction of systemic resistance and subsequent upregulation of the plant defense-linked genes [7,9,20,21].

The purpose of this study was to identify antifungal genes in the genome of the bacterial strains isolated from cotton roots and mycelial deformities in VD-080 induced by the bacterial extracts and to understand the key role of the bacterial strains in the suppression of *V. dahliae* plus elicitation of defense response of cotton plants against the pathogen through modulation of defense-linked genes, viz., *HMGR*, *MPK3*, *GST*, *PAL*, *PPO* and *SOD*.

## 2. Results

### 2.1. Identification and Phylogenetic Analysis of Bacterial Endophytes

**Figure 1:**
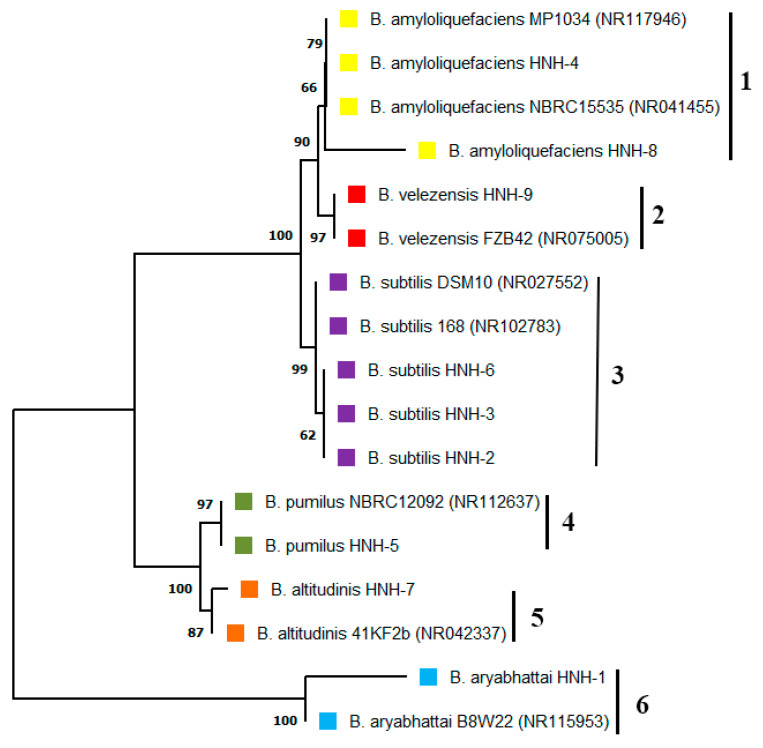
Phylogenetic studies of endophytic *Bacillus* strains isolated from cotton roots. The optimal neighbor joining tree indicating that the bacterial endophytes fall into six different clusters. The numbers added with each branch indicate the species, where 1 = 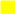 *B. amyloliquefaciens*, 2 = 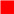 *B. velezensis*, 3 = 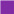 *B. subtilis*, 4 = 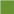 *B. pumilus*, 5 = 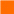 *B. altitudinis*, 6 = 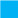 *B. aryabhattai*.

The endophytic bacterial strains isolated from cotton roots were identified through PCR analysis and subsequent 16S rDNA gene sequencing. The results indicated that HNH2, HNH3 and HNH6 had 99.86%, 99.80% and 99.86% resemblance to *B. subtilis*. The isolates HNH4 and HNH8 showed 99.93% and 99.86% sequence homology to *B. amyloliquefaciens*. Moreover, HNH1, HNH5, HNH7 and HNH9 were 99.80%, 99.92%, 100% and 100% identical to *B. aryabhattai*, *B. pumilus*, *B.altitudinis* and *B. velezensis*, respectively. A neighbor joining phylogenetic tree constructed based on the 16S rDNA gene sequence indicated that these bacterial endophytes fall into six different clusters of *B. amyloliquefaciens*, *B. subtilis*, *B. velezensis*, *B. pumilis*, *B. altitudinis* and *B. aryabhattai* (Figure 1). The results indicated that many kinds of *Bacillius* strains colonized in the roots of upland cotton plants.

### 2.2. Antagonistic Activity of the Bacterial Isolates against VD-080

**Figure 2:**
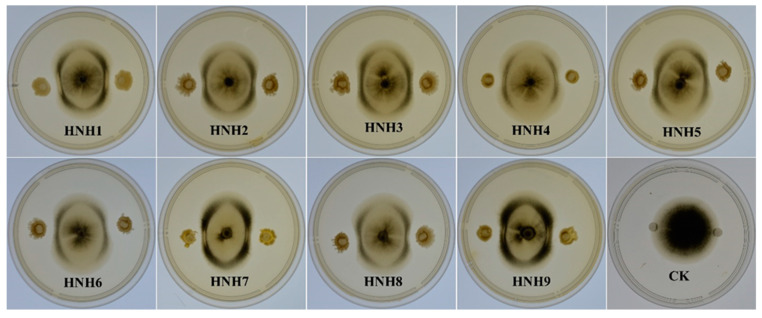
Antagonistic effect of endophytic *Bacillus* strains against VD-080.

Nine bacterial endophytes isolated from cotton roots were evaluated for their antagonistic activity against VD-080 in a dual culture assay. Results showed that all the isolated endophytic *Bacillus* strains were able to inhibit the growth of VD-080, but *B. altitudinis* HNH7 and *B. velezensis* HNH9 showed the highest inhibition of fungal growth. The diameter of inhibition zones was 10.5 and 9.3 mm for HNH7 and HNH9, respectively (Table 1). The results showed that the nine endophytic *Bacillius* strains could devour *V. dahliae* (VD-080), with variable antagonistic effects (Figure 2).

### 2.3. Interaction of Extracted Lipopeptides (LPs) with VD-080

**Figure 3:**
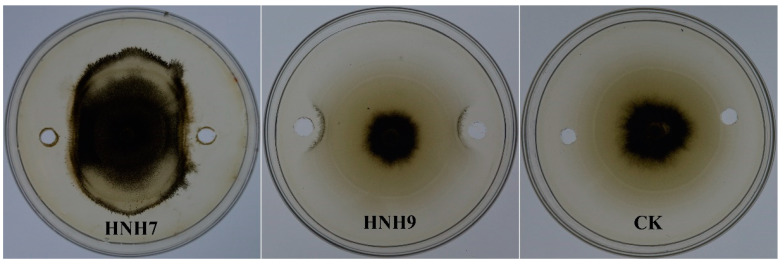
Antifungal effect of crude extracts from endophytic *Bacillus* strains HNH7 and HNH9 against VD-080.

Methanolic extracts (LPs) of bacterial strains performing best in dual culture assay were evaluated for their antifungal activity against VD-080. Results demonstrated that the methanolic extracts of both endophytic strains HNH7 and HNH9 significantly inhibited the hyphal growth of VD-080 compared to control. The growth restriction of the pathogenic fungi around the holes containing crude extracts of bacterial LPs can be observed easily, while the holes of only methanol in the control plate are fully covered by the fungal mycelium (Figure 3).

### 2.4. PCR-Based Detection of LPs Encoded Genes

**Figure 4:**
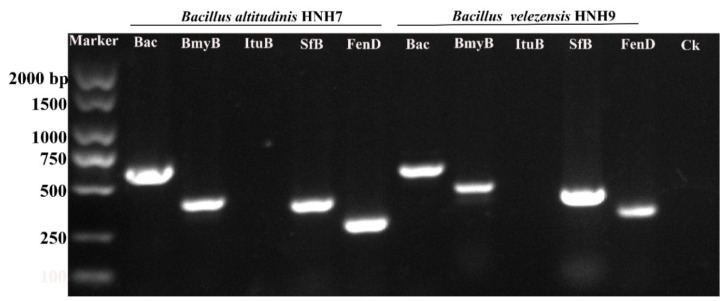
Genetic marker-assisted detection of antimicrobial compounds synthetase genes in HNH7 and HNH9. Bac = Bacillibactin, BmyB = Bacillomycin, ItuB = Iturin, SfB = Surfactin and FenD = Fengycin. Ck = Control.

The genes encoding specific antimicrobial compounds, viz., surfactin (SfB), iturin (ItuB), bacillomycin (BmyB), bacillibactin (Bac) and fengycin (FenD), were amplified through PCR using gene-specific molecular markers (Figure 4). The results of PCR amplification revealed that both HNH7 and HNH9 possessed surfactin, bacillomycin, bacillibactin and fengycin encoding genes simultaneously. However, the iturin encoding gene ItuB was not detected in both bacterial strains. Bacillibactin encoding gene yielded a band of 595 bp, while Bacillomycin, surfactin and fengycin yielded bands between 230 and 400 bp.

### 2.5. LC-MS Analysis of Antimicrobial Compounds

**Figure 5:**
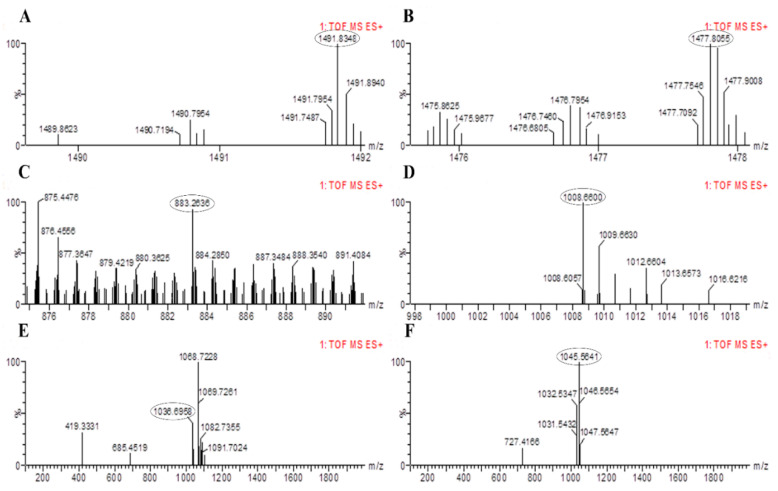
LC-MS chromatogram of methanolic extracts of *B. altitudinis* HNH7. (**A**,**B**) = Fengycin; (**C**) = Bacillibactin; (**D**,**E**)= Surfactin and (**F**) = Bacillomycin.

**Figure 6:**
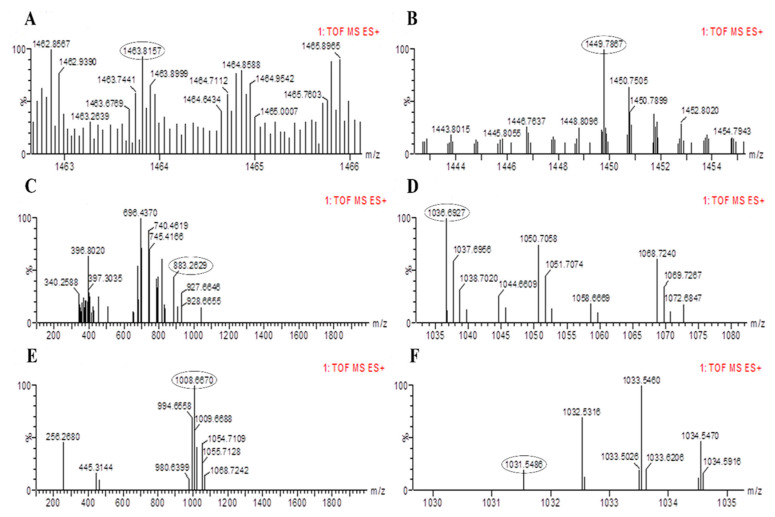
LC-MS chromatogram of methanolic extracts of *B. amyloliquefaciens* HNH9. (**A**,**B**) = Fengycin, (**C**) = Bacillibactin, (**D**,**E**)= Surfactin and (**F**)= Bacillomycin.

The results of PCR amplification of antimicrobial genes were further validated through LC-MS analysis. The LC-MS results confirmed that both bacterial strains were able to produce corresponding products of the antimicrobial genes identified based on their mass to charge ratio (m/z). The peaks for fengycin were detected in *B. altitudinis* HNH7 at 1491.83 and 1477.80 m/z (Figure 5); however, it was detected at 1463.81 and 1449.78 m/z in the case of *B. velezensis* HNH9 (Figure 6). Bacillibactin was observed at 883.26 m/z in both the endophytes. Surfactin was detected at 1008.66 and 1038.69 m/z in both the endophytes. Further, Bacillomycin was detected at 1045.56 and 1031.54 m/z in HNH7 and HNH9, respectively. These detected antimicrobial substances explain a clear signal of the endophytes being antagonistic to VD-080.

### 2.6. Hyphal Anomalies in VD-080 by Methanolic Extracts of Endophytic Bacillus Strains

**Figure 7:**
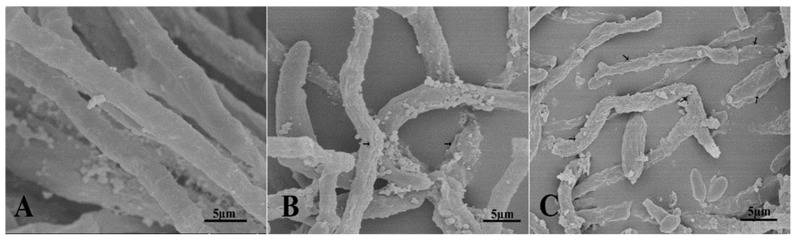
Ultrastructural changes induced by methanolic extracts of endophytic *Bacillus* strains HNH7 and HNH9; (**A**) = Control; (**B**) = HNH9 and (**C**) = HNH7.

The structural deformities in VD-080 hyphae caused by the antimicrobial compounds produced by both endophytic *Bacillus* strains were studied under scanning electron microscope (Figure 7). The hyphae treated with methanolic extracts of HNH7 and HNH9 strains showed pore formation, breakdown of hyphae, curling, shirking and removal of cytoplasmic content from the hyphae. Meanwhile, the hyphae in the control treatment were long, cylindrical and healthy with no structural abnormalities. The results indicated that the LPs of the *Bacillius* strains could damage the cell structure of VD-080.

### 2.7. Biocontrol Effects of the Isolates on Cotton Verticillium Wilt

**Figure 8:**
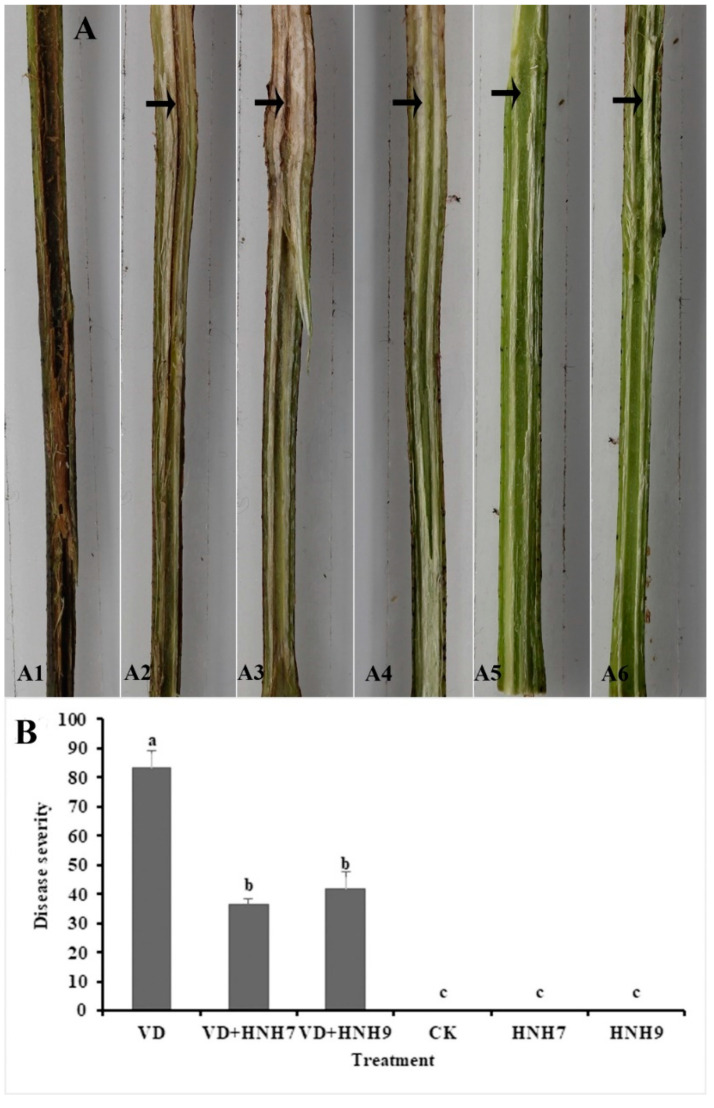
Greenhouse evaluation of disease incidence reduction by endophytic bacterial strains in cotton plants. A1 = VD-080 only, A1 = HNH7 + VD-080, A3 = HNH9 + VD-080, A4 = Ck (water only), A5 = HNH7 and A6 = HNH9. (**A**) = Infection progress in cotton stem, (**B**) = disease severity. → indicates the infected part of the plant. The disease severity index and biocontrol efficacy were recorded on a 0–4 scale, where 0 = no symptoms or no vascular browning; 1 = 1–25% vascular browning; 2 = 26–50% vascular browning; 3 = 51–75% vascular browning; 4 = more than 75%.The mean values were computed and LSD test at *p* ≤ 0.05 after one-way ANOVA was conducted. The error bars indicate the standard error ± SE of the mean values. Corresponding results of ANOVA, F = 97.9, *p* = 0.000. Letters (a–c) above the columns represent significant differences between treatment means at *p* ≤ 0.05.

The ability of endophytic bacterial strains to inhibit verticillium wilt disease in cotton plants was evaluated in a greenhouse experiment. Disease severity was recorded 30 days post-inoculation by observing vascular browning. The highest disease severity (83.33%) was observed in the treatment which was inoculated with VD-080 only. Furthermore, there was a significant reduction in disease severity in treatments which were administered with endophytic bacterial strains HNH7 and HNH9 and challenged with VD-080 compared to the treatments inoculated with VD-080 only. HNH7 showed higher reduction of disease than HNH9 as the disease severity recorded was 36.55% by HNH7 and 42.08% by HNH9 compared to control (Figure 8).

### 2.8. Expression Profiling of Plant Defense-Linked Genes

**Figure 9:**
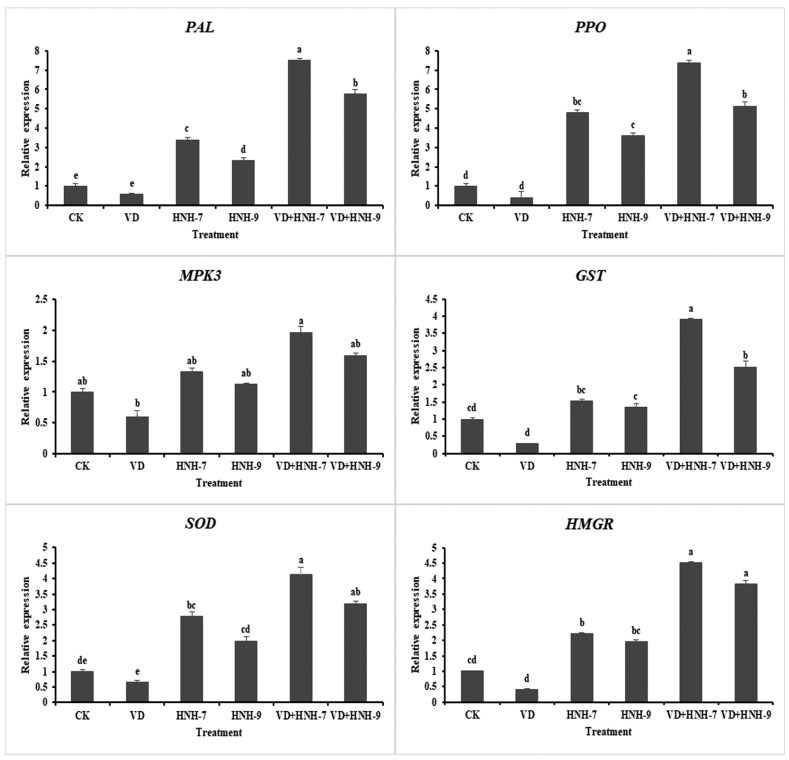
Expression profiling of 6 defense-linked genes in cotton was upregulated in treatments with the isolates. The mean values were computed and separated by LSD test at *p* ≤ 0.05 after one-way ANOVA. The error bars indicate the standard error ± SE of the mean values. Letters above the columns (a–e) represent significant differences between treatment means at *p* ≤ 0.05.

The expression profiling of six defense-linked genes, viz., *MPK3*, *GST*, *SOD*, *PAL*, *PPO* and *HMGR*, in cotton plants was studied through qRT-PCR. According to the results, both bacterial endophytes elicited the defense response in cotton plants as the expression of defense-linked genes in plants treated with bacterial endophytes was significantly higher compared to the plants inoculated with water only. Interestingly, the expression of defense-linked genes was much higher in plants treated with bacterial antagonists and challenged with VD-080 also. However, the expression of genes was higher in the plants treated with endophytic bacterial strains HNH7 compared to HNH9. The highest expression was recorded in the case of *PAL* and *PPO* encoding genes. Moreover, the expression of all defense-linked genes was found to be downregulated in the plants challenged with the pathogen only (Figure 9).

## 3. Discussion

The plants endophytes of *Pseudomonas, Serratia* and *Bacillus* spp. have been analyzed as efficient biocontrol agents against verticillium wilt both in vitro and in vivo [7,22]. The plant root colonized bacteria apply different built-in tools to initiate defense mechanisms which progressively devour the pathogenic fungi. The potential of plant growth promoting rhizobacteria (PGPRs) to produce hydrolytic enzymes, siderophores, a range of different antibiotics, aggressive colonization and induced systemic resistance, which are the basic mechanisms to prevent the proliferation of plant pathogens [23]. However, endophyte-allied antibiotics and ISR have positive effects on competitive interactions with plants and the plant pathogens which inhibit the synthesis of pathogen cell walls and cell membrane structures and disrupt ribosomal subunits [24]. The bacterial strains 41B-1 and SQR9 have shown significant biocontrol activity against *V. dahliae* in greenhouse and field trials [7,25].

In this study, we characterized nine endophytic *Bacillus* strains isolated from cotton roots. These endophytic strains include *B. amyloliquefaciens*, *B. subtilis*, *B. velezensis*, *B. pumilis*, *B. altitudinis* and *B. aryabhattai*. All these strains show variable antagonistic effects to VD-080. Two of the endophytes, *B. altitudinis* (HNH7) and *B. velezensis* (HNH9), exhibited high biocontrol efficacy. The composition of endophytic strains which have antifungal effects on *V. dahliae* may be related to bacterial antibiotics and induce resistance in cotton cultivars against verticillium wilt. Endophytic bacteria have shown antagonistic activity against several phytopathogens such as *Fusarium oxysporum* f. sp. *cucumerinum*, *Rhizoctonia solani*, *F. oxysporum* f. sp. *niveum*, *F. oxysporum* f. sp. *cubense* and *V. dahliae* [22,26,27].

*Bacillus* species can produce several antibiotics such as surfactin, fengycin, bacilysin, bacillibactin, bacillomycin and iturin [28,29]. The antimicrobial compounds produced by *Bacillus* species either directly hamper the growth of the pathogen or induce systemic resistance in plants [19,30]. The isolates HNH7 and HNH9 were found to possess bacillibactin, bacillomycin, surfactin, and fengycin encoding genes, while both were lacking in iturin producing genes. One siderophore producing (bacillibactin), two antifungal (bacillomycin and fengycin) and one multifunctional (antifungal plus antibacterial) metabolite (surfactin) encoding genes were successfully detected in the genome of HNH7 and HNH9. Fengycin, surfactin, bacillibactin and bacillomycin have been reported previously to exhibit antagonistic activity against fungal pathogens including *V. dahliae* [7], which is consistent with our LC-MS results, where the antifungal substances were detected through LC-MS at their specific m/z values (fengycin at m/z 1463.81, 1449.78, 1491.83, 1477.80; bacillibactin at 883.26; surfactin at 1036.69, 1008.66, 1068.72 and bacillomycin at 1031.54, 1045.56). Conclusively, the coproduction of these antimicrobial compound producing genes and gene products have a possible cumulative role in antagonism against *V. dahliae* (VD-080).

Microbial lipopeptides (LPs) can cause morphological and ultrastructural deformities in fungal hyphae such as pore formation, curling, plasmolysis or removal of cytoplasmic content and breakdown or disintegration of fungal hyphae [19,31,32,33]. Scanning electron microscopy (SEM) analysis has previously supported the hypothesis that the loss of turgidity and structural alterations in the cell wall of fungal hyphae is the core mechanism involved in the antifungal action of LPs [32,33]. In our study, the SEM analysis revealed disruption of *V. dahliae* (VD-080) hyphae when treated with the LPs of HNH7 and HNH9 compared to control treatment and these morphological changes in the hyphae explain the possible deleterious role of LPs produced by the two endophytes.

*Bacillus* spp. protect plants against phytopathogens through the induction of systemic resistance and subsequent upregulation of defense-linked genes [7,9,20,21]. Our study demonstrated that the disease severity on cotton plants treated with endophytes was significantly reduced compared to the infected control treatment, plus the expression of defense-linked genes *HMGR*, *MPK3*, *GST*, *PAL*, *PPO* and *SOD* was significantly upregulated in cotton plants treated with endophytes + VD-080, compared to the treatments inoculated with HNH7 and HNH9. Interestingly, the activities of defense-linked genes were higher in HNH-7 and HNH-9 treated plants compared to controls, indicating that the endophytes were responsible for defense elicitation in cotton plants. Our results are in conformation with the findings of Chandrasekaran and Chun [34], where the expression of defense-linked genes *SOD*, *CAT*, *POD*, *PPO*, *PAL* and *β-1,3-glucanase* was significantly increased in *Bacillus subtilis* + pathogen-treated tomato plants. SOD is responsible for H_2_O_2_ accumulation in plants. According to several previous studies, H_2_O_2_ has a significant role in disease resistance against pathogens [35,36]. It can be concluded that SOD expression induced by endophytes provided protection against VD-080. PAL contributes to the oxidation of phenolic compounds and defense stimulation against the pathogens [35]. In addition to SOD and PAL, the activity of PPO was also upregulated in our study. According to Li and Steffens [37], the overexpression of PPO in the plants leads to enhanced resistance against the disease.

The high expression of *MPK3*, *HMGR* and *GST* genes in the present study indicated their possible role in defense elicitation. These genes and their corresponding products play several important roles in the plant defense system. Mitogen-activated protein kinases (MPK) are a cascade of signaling molecules that play an important role in signal transduction to activate plant defense against the pathogens [38,39]. According to a previous study, the enhanced expression of *MPK3* gene has been involved in providing basal resistance against *Botrytis cinerea* [40]. Glutathione S-transferase (GST) is an enzyme with detoxifying properties that is responsible for stress modulation in plants and provides increased resistance against several biotic and abiotic stresses [41,42]. HMG-CoA reductase (3-hydroxy-3-methyl-glutaryl-coenzyme A reductase) *HMGR* is an enzyme that catalyzes the first step in the mevalonate (MVA) pathway responsible for the formation of isoprenoid [43,44]. Isoprenoids are significant in the elicitation of plant defense against biotic and abiotic stresses.

Based on the present study and previous reports, we suggest that the enhanced expression of defense-related genes is responsible for the strong biological control activity of HNH-7 and HNH-9 and plays a vital role in plant defense against *Verticillium dahliae*.

## 4. Materials and Methods

### 4.1. Isolation of Endophytic Bacterial Isolates

Endophytic *Bacillus* strains were isolated from cotton roots following the procedure given by Khan et al. [45]. Briefly, cotton roots of cultivar “Jimian 11”, grown at Liuhe Experimental Station, Nanjing, Jiangsu Province, were washed with tap water, excised into small pieces of 5 mm, surface sterilized with 2.5% sodium hypochlorite (NaClO) for 10 min and then rinsed four times with double distilled sterile water to remove the contaminants. Afterwards, the root was ground in 1 mL of double distilled water using mortar and pestle. Post-grinding, an aliquot of 200 µL was spread onto LB agar plate and incubated at 37 ± 2 °C for 12 h. Post-incubation, the bacterial colonies were observed and the colonies differing in size and shape were selected, picked and cultured in LB medium.

### 4.2. 16S rDNA Identification

The endophytic bacterial isolates were characterized by sequencing the 16S rDNA gene [46]. DNA of endophytic isolates was extracted by using DNA extraction kit (Omega Bio-tek, Norcross, GA, USA). The concentration and purity of extracted DNA was recorded on NanoDrop 1000 (Thermo Scientific, Wilmington, DE, USA). The 16s rDNA primers forward 531F (5′-TGGAGAGTTTGATCCTGGCTCAG-3′) and reverse 531R (5′-TACCGCGGCTGCTGGCAC-3′) were used for amplification of the 16S rDNA gene in bacterial endophytes. PCR amplification was processed using 2× Rapid Taq Master Mix (Vazyme Biotech Co.,Ltd, Nanjing, China). The reaction conditions were as follows: initial denaturation at 95 °C for 5 min, followed by 32 cycles of denaturation at 95 °C for 15 sec, annealing at varied temperatures according to the set of primers, elongation at 72 °C for 15 sec and, finally, a cycle of final extension for 5 min at 72 °C. Additionally, a blank (CK) containing sterilized distilled water rather than genomic DNA was also included in the PCR reaction. The amplified product (5 µL) was visualized on 1% agarose gel stained with ethidium bromide and remaining product was sequenced using the Sanger dideoxy sequencing method (Genscript Co. Ltd. Nanjing, Jiangsu, China). The obtained sequences were compared with the previously available sequences in the NCBI database and the phylogenetic relationship was studied [47]. The homologous sequences were analyzed using MEGA X software [48]. Multiple sequence alignments of the sequences were performed by ClustalW and a neighbor joining phylogenetic tree was constructed using bootstrap test (1000 replicates) and evolutionary distances were calculated using Tamura Nei model [49]. A gamma distribution (shape parameter = 5) model was used to determine the rate variation among sites with partial deletion of gaps and missing data.

### 4.3. Antagonistic Activity of Endophytes against V. dahliae (VD-080)

The endophytic bacterial isolates were evaluated for their inhibitory effect against VD-080 in a dual culture experiment [7]. The fungus was inoculated at the center of the Petri plate (9 cm) containing Potato dextrose agar medium (PDA) and incubated for 3 days at 27 ± 2 °C. Endophytic bacterial isolates (5 µL) from an overnight culture (OD600 = 2.5) were inoculated on two sides of the fungal colony 2.5 cm away from the center. The plates were then wrapped with a parafilm and incubated at 27 ± 2 °C for seven days and the zone of inhibition was measured. The experiment was repeated thrice with three replicates.

### 4.4. Antifungal Activity of Secondary Metabolites

The bacterial strains HNH7 and HNH9 were individually inoculated in Landy medium [50] from an overnight culture of each strain and incubated for 3 days at 30 °C and 180 rpm. Post-incubation, the cultures were centrifuged at 10,000 rpm and 4 °C for 15 min. The cell free supernatants were collected in new tubes and each supernatant was incubated at 4 °C for 12 h after adjusting pH to 2 by adding concentrated HCl. Afterwards, the precipitates were collected through centrifugation and dissolved in 5 mL of HPLC grade methanol [51]. The crude extract of bacterial secondary metabolites (pH 7.0) was passed through 0.22 µm syringe filter to remove impurities and used for the determination of antifungal activity against VD-080. Fungus was grown on PDA for two days and then 10 µL of bacterial crude extract was pipetted in two sides 2.5 cm away from the fungus. In control treatment, methanol was used instead of crude extract.

### 4.5. Identification of Antifungal Genes Using Molecular Markers

Molecular markers reported by Farzand et al. [15] were used for amplification of genes, viz., iturin, bacillomycin, surfactin, bacillibactin and fengycin, responsible for producing corresponding antimicrobial secondary metabolites (Table 2). These genes were amplified through PCR in a thermal cycler (Bio-Rad, Hercules, CA, USA).

### 4.6. LC-MS Analysis of Antimicrobial Compounds

The crude extract of bacterial endophytes was passed through a 0.22 µm syringe filter and used for the detection of antimicrobial compounds. A surveyor LC-MS-system G2 QTof-XS, a Waters (Santa Clara, CA, USA) was employed following the protocol described by Hajji et al. [52].

### 4.7. Scanning Electron Microscopy

Crude extract of endophytic bacterial isolates was evaluated for its ability to cause structural deformities in fungal mycelium through scanning electron microscope following the protocol of Gu et al. [53]. *V. dahliae* (VD-080) was cultured for 3 days at 25 °C and 50 rpm in potato dextrose broth. Afterward, the hyphae were harvested in 2 mL Eppendorf tube and treated with crude methanolic extract (50 µg/mL) of HNH7 and HNH9 for 12 h at 27 ± 2 °C and 180 rpm. Hyphae in the control treatment were treated with HPLC grade methanol only. Furthermore, the Eppendorf tubes were centrifuged, and the hyphae were washed three times with 1× PBS buffer. Finally, the hyphae were preserved in 2.5% (*v*/*v*) glutaraldehyde solution. Structural abnormalities of VD-080 were observed through scanning electron microscope [31].

### 4.8. Biocontrol Effects of Endophytes in Greenhouse Experiment

The seeds of susceptible cotton cultivar cv. “Jimian11” were surface disinfected with 1.5% NaOCl solution for 3 min and sown in a mixture of soil containing sterilized peat and vermiculite 1:1 (*v*/*v*). The experiment comprised six treatments: (1) VD-080 only, (2) co-inoculated with HNH7 + VD-080, (3) co-inoculated with HNH9 + VD-080, (4) CK (water only), (5) HNH7 only and (6) HNH9 only. Each treatment was replicated 3 times. The plants were treated with 20 mL bacterial cell suspension (OD600 = 2.5) (1 × 10^8^ cfu/mL) at four-leaf stage following the method given by Idris et al. [54]. Furthermore, 24 h after treating the plants with bacterial cell suspension, the roots of plants in respective treatments were inoculated with VD-080 conidial suspension (1 × 10^7^ cfu/mL). Thirty days post-inoculation, the disease severity in plants was recorded following the vascular browning scale given by Bawa et al. [55]. The disease severity index and biocontrol efficacy were calculated by cutting the basal stem vertically and vascular browning was recorded on a 0–4 scale, where 0 = no symptoms or no vascular browning; 1 = 1–25% vascular browning; 2 = 26–50% vascular browning; 3 = 51–75% vascular browning; 4 = more than 75% vascular browning, and calculated using the following formula. Disease severity index (DSI) = [(A × 0) + (B × 1) + (C × 2) + (D × 3) + (E × 4)]/M × 4, where A, B, C, D and E = number of plants with rating scales of 0, 1, 2, 3 and 4, respectively, while M = total number of plants in the treatment observed.

### 4.9. Expression Analysis of Defense-Linked Genes in Cotton Plants through qRT-PCR

The expression of defense-linked genes in cotton plants (Table 3) was studied through qRT-PCR. Cotton leaves from each treatment were used to extract total RNA using total plant RNA extraction kit (Omega Bio-Tek, Norcross, GA, USA). NanoDrop 1000 (Thermo Scientific, Wilmington, DE, USA) was employed to measure the concentration and purity of extracted RNA. First-strand cDNA was synthesized using Evo M-MLV reverse transcriptase (Accurate Biology, Hunan, China). The expression of defense-linked genes, viz., *HMGR*, *MPK3*, *GST*, *PAL*, *PPO* and *SOD*, was studied using the SYBR Green Premix Taq HS qPCR kit (Accurate Biology, Hunan, China) in qRT-PCR (QuantStudio-6, California, USA). Sequences of defense-linked genes were obtained from NCBI [56].. The expression of genes was normalized by using actin as a housekeeping gene. Final expression of the target genes was quantified in three repeats using 2^−ΔΔCt^ method derived from Livak and Schmittgen [57].

### 4.10. Statistical Analysis

All the experiments were carried out in a completely randomized design (CRD) and repeated thrice. Data were subjected to one-way ANOVA using SPSS software. The treatment means were compared and separated using LSD test at *p* ≤ 0.05.

## 5. Conclusions

Conclusively, the cotton endophytes *B. altitudinis* and *B. velezensis* (HNH7 and HNH9) possessed the powerful capabilities to synthesize different kinds of antimicrobial metabolites and elicitation of ISR to show strong antagonistic activities against *Verticillium dahliae*. Moreover, transcriptional profiling of defense-linked genes, viz., *HMGR*, *MPK3*, *GST*, *PAL*, *PPO* and *SOD*, through RT-qPCR showed induction of the plant defense mechanism. These results indicated that interactions between HNH7 and HNH9 provide efficient antibiotic-mediated inhibition of the VD-080 pathogen. These strains can be used to formulate biopesticides in the future for controlling verticillium wilt disease.

## Figures and Tables

**Table 1:** Mycelial inhibition by endophytic *Bacillus* strains.

Endophytic *Bacillus* Strains	Inhibition Zone (mm)
*B. aryabhattai* HNH1	7.1 ± 0.11 d
*B. subtilis* HNH2	7.0 ± 0.10 d
*B. subtilis* HNH3	7.7 ± 0.10 c
*B. amyloliquefaciens* HNH4	7.6 ± 0.06 c
*B pumilus* HNH5	5.0 ± 0.09 f
*B. subtilis* HNH6	6.9 ± 0.07 d
*B. altitudinis* HNH7	10.5 ± 0.07 a
*B. amyloliquefaciens* HNH8	5.9 ± 0.09 e
*B. velezensis* HNH9	9.3 ± 0.09 b
*CK	0.0 ± 0.00 g

Each value represents a mean ± SE of three replicates. *CK = control. Corresponding results of ANOVA. F = 1161, *p* = 0.000, dF = 9. Values in the columns followed by the same letters (a–g) are not significantly different from each other according to LSD test (*p* ≤ 0.05).

**Table 2:** Genetic markers used for the detection of antifungal genes in genomes of the isolates.

Product	Gene	Temp (°C)	Primer Sequences	Size (bp)	Reference
Bacillibectin	BAC	57.6	ATCTTTATGGCGGCAGTC ATACGGCTTACAGGCGAG	595	[15]
Bacillomycin	BMYB	55.3	CGAAACGACGGTATGAAT TCTGCCGTTCCTTATCTC	371	[15]
Iturin	ITUB	55.1	ATCACCGATTCGATTTCA GCTCGCTCCATATTATTTC	708	[15]
Surfactin	SFB	50.0	TTCACACAATTAGAGCT ATATGATGATTGCTCCAG	338	[15]
Fengycin	FEND	57.6	TCAGCCGGTCTGTTGAAG TCCTGCAGAAGGAGAAGT	231	[15]

**Table 3:** Oligonucleotides used in this study for qRT-PCR analysis.

No.	Name	Code	Primers (5′ to 3′)
1	3-hydroxy-3-methylglutaryl-coenzyme A reductase	*HMGR*-F	GTTACAACCGAGGAAGACGAG
		*HMGR*-R	CAATGGCAAACCCGATAACG
2	Mitogen-activated protein kinase 3	*MPK3*-F	AAATACCCTAAGCCATCCACC
		*MPK3*-R	CCAACCCAATTCCCATTTGTG
3	Glutathione-S-transferase	*GST*-F	TCAGTGCTTTCCTACCCTTTG
		*GST*-R	ATACCCAACAGAGCTAGCAAC
4	Phenylalanine ammonia-lyase	*PAL*-F	ATGTTTGCTCAGTTTTCGGAAC
		*PAL*-R	GGCACTTTGAACATGGTTGG
5	Polyphenol oxidase	*PPO*-F	GAGTCAAGGTTCGTGATAGCC
		*PPO*-R	GGTGATGTTCTTTGTTTCGGC
6	Superoxide dismutase	*SOD*-F	CTGCCTCTGTCTCGATCATTG
		*SOD*-R	ACCTTTCTGAATAGCCTCATGG
7	Actin	*Actin*-F	CGAGACATTGGGACAGGTATTG
		*Actin*-R	GAGATCACGACCAGCAAGG

## References

[1] Wang Y., Liang C., Wu S., Zhang X., Tang J., Jian G., Jiao G., Li F., Chu C. (2016). Significant improvement of cotton Verticillium wilt resistance by manipulating the expression of Gastrodia antifungal proteins. Mol. Plant.

[2] Zhang K., Pei Z., Hongmei W., Yunlei Z., Wei C., Haiyan G., Xiaohui S., Yanli C. (2019). Isolation and characterization of the GbVIP1 gene and response to Verticillium wilt in cotton and tobacco. J. Cotton Res..

[3] Goicoechea N. (2009). To what extent are soil amendments useful to control Verticillium wilt?. Pest Manag. Sci. Former. Pestic. Sci..

[4] Berg G., Hallmann J. (2006). Control of plant pathogenic fungi with bacterial endophytes. Microbial Root Endophytes.

[5] Chen L., Shi H., Heng J., Wang D., Bian K. (2019). Antimicrobial, plant growth-promoting and genomic properties of the peanut endophyte Bacillus velezensis LDO2. Microbiol. Res..

[6] Han Q., Wu F., Wang X., Qi H., Shi L., Ren A., Liu Q., Zhao M., Tang C. (2015). The bacterial lipopeptide iturins induce V erticillium dahliae cell death by affecting fungal signalling pathways and mediate plant defence responses involved in pathogen-associated molecular pattern-triggered immunity. Environ. Microbiol..

[7] Li B., Li Q., Xu Z., Zhang N., Shen Q., Zhang R. (2014). Responses of beneficial Bacillus amyloliquefaciens SQR9 to different soilborne fungal pathogens through the alteration of antifungal compounds production. Front. Microbiol..

[8] Li C.H., Shi L., Han Q., Hu H.L., Zhao M.W., Tang C.M., Li S.P. (2012). Biocontrol of Verticillium wilt and colonization of cotton plants by an endophytic bacterial isolate. J. Appl. Microbiol..

[9] Zhang F., Li X.-L., Zhu S.-J., Ojaghian M.R., Zhang J.-Z. (2018). Biocontrol potential of Paenibacillus polymyxa against Verticillium dahliae infecting cotton plants. Biol. Control.

[10] Hanif A., Zhang F., Li P., Li C., Xu Y., Zubair M., Zhang M., Jia D., Zhao X., Liang J. (2019). Fengycin produced by Bacillus amyloliquefaciens FZB42 inhibits Fusarium graminearum growth and mycotoxins biosynthesis. Toxins.

[11] Hussein W., Awad H., Fahim S. (2016). Systemic resistance induction of tomato plants against ToMV virus by surfactin produced from Bacillus subtilis BMG02. Am. J. Microbiol. Res..

[12] Lu X., Zhou D., Chen X., Zhang J., Huang H., Wei L. (2017). Isolation and characterization of Bacillus altitudinis JSCX-1 as a new potential biocontrol agent against Phytophthora sojae in soybean [Glycine max (L.) Merr.]. Plant Soil.

[13] Nicholson W. (2002). Roles of Bacillus endospores in the environment. Cell. Mol. Life Sci. CMLS.

[14] Compant S., Duffy B., Nowak J., Clément C., Barka E.A. (2005). Use of plant growth-promoting bacteria for biocontrol of plant diseases: Principles, mechanisms of action, and future prospects. Appl. Environ. Microbiol..

[15] Farzand A., Moosa A., Zubair M., Khan A.R., Hanif A., Tahir H.A.S., Gao X. (2019). Marker assisted detection and LC-MS analysis of antimicrobial compounds in different Bacillus strains and their antifungal effect on Sclerotinia sclerotiorum. Biol. Control.

[16] Koumoutsi A., Chen X.-H., Henne A., Liesegang H., Hitzeroth G., Franke P., Vater J., Borriss R. (2004). Structural and functional characterization of gene clusters directing nonribosomal synthesis of bioactive cyclic lipopeptides in Bacillus amyloliquefaciens strain FZB42. J. Bacteriol..

[17] Chen X.-H., Vater J., Piel J., Franke P., Scholz R., Schneider K., Koumoutsi A., Hitzeroth G., Grammel N., Strittmatter A.W. (2006). Structural and functional characterization of three polyketide synthase gene clusters in Bacillus amyloliquefaciens FZB 42. J. Bacteriol..

[18] Duan C., Yu J., Bai J., Zhu Z., Wang X. (2014). Induced defense responses in rice plants against small brown planthopper infestation. Crop J..

[19] Farzand A., Moosa A., Zubair M., Khan A.R., Massawe V.C., Tahir H.A.S., Sheikh T.M.M., Ayaz M., Gao X. (2019). Suppression of Sclerotinia sclerotiorum by the Induction of Systemic Resistance and Regulation of Antioxidant Pathways in Tomato Using Fengycin Produced by Bacillus amyloliquefaciens FZB42. Biomolecules.

[20] Rais A., Jabeen Z., Shair F., Hafeez F.Y., Hassan M.N. (2017). Bacillus spp., a bio-control agent enhances the activity of antioxidant defense enzymes in rice against Pyricularia oryzae. PLoS ONE.

[21] Sahu P.K., Singh S., Gupta A., Singh U.B., Brahmaprakash G., Saxena A.K. (2019). Antagonistic potential of bacterial endophytes and induction of systemic resistance against collar rot pathogen Sclerotium rolfsii in tomato. Biol. Control.

[22] López-Escudero F.J., Mercado-Blanco J. (2011). Verticillium wilt of olive: A case study to implement an integrated strategy to control a soil-borne pathogen. Plant Soil.

[23] Beneduzi A., Ambrosini A., Passaglia L.M. (2012). Plant growth-promoting rhizobacteria (PGPR): Their potential as antagonists and biocontrol agents. Genet. Mol. Biol..

[24] Maksimov I., Abizgil’Dina R., Pusenkova L. (2011). Plant growth promoting rhizobacteria as alternative to chemical crop protectors from pathogens. Appl. Biochem. Microbiol..

[25] Qin Y., Han Y., Yu Y., Shang Q., Zhang B., Li P. (2015). Complete genome sequence of Bacillus amyloliquefaciens L-S60, a plant growth-promoting and antifungal bacterium. J. Biotechnol..

[26] Massawe V.C., Hanif A., Farzand A., Mburu D.K., Ochola S.O., Wu L., Tahir H.A.S., Gu Q., Wu H., Gao X. (2018). Volatile compounds of endophytic Bacillus spp. have biocontrol activity against Sclerotinia sclerotiorum. Phytopathology.

[27] Milijašević-Marčić S., Todorović V., Stanojević O., Berić T., Stanković S., Todorović B., Potočnik I. (2018). Antagonistic potential of Bacillus spp. isolates against bacterial pathogens of tomato and fungal pathogen of pepper. Pestic. I Fitomedicina.

[28] Joshi R., McSpadden Gardener B.B. (2006). Identification and characterization of novel genetic markers associated with biological control activities in Bacillus subtilis. Phytopathology.

[29] Stein T. (2005). Bacillus subtilis antibiotics: Structures, syntheses and specific functions. Mol. Microbiol..

[30] Romero-Tabarez M., Jansen R., Sylla M., Lünsdorf H., Häußler S., Santosa D.A., Timmis K.N., Molinari G. (2006). 7-O-malonyl macrolactin A, a new macrolactin antibiotic from Bacillus subtilis active against methicillin-resistant Staphylococcus aureus, vancomycin-resistant enterococci, and a small-colony variant of Burkholderia cepacia. Antimicrob. Agents Chemother..

[31] Farzand A., Moosa A., Zubair M., Khan A.R., Ayaz M., Massawe V.C., Gao X. (2020). Transcriptional Profiling of Diffusible Lipopeptides and Fungal Virulence Genes During Bacillus amyloliquefaciens EZ1509-Mediated Suppression of Sclerotinia sclerotiorum. Phytopathology.

[32] Romero D., De Vicente A., Olmos J., Dávila J., Pérez-García A. (2007). Effect of lipopeptides of antagonistic strains of Bacillus subtilis on the morphology and ultrastructure of the cucurbit fungal pathogen Podosphaera fusca. J. Appl. Microbiol..

[33] Vitullo D., Di Pietro A., Romano A., Lanzotti V., Lima G. (2012). Role of new bacterial surfactins in the antifungal interaction between Bacillus amyloliquefaciens and Fusarium oxysporum. Plant Pathol..

[34] Chandrasekaran M., Chun S.C. (2016). Expression of PR-protein genes and induction of defense-related enzymes by Bacillus subtilis CBR05 in tomato (Solanum lycopersicum) plants challenged with Erwinia carotovora subsp. carotovora. Biosci. Biotechnol. Biochem..

[35] Li Y., Gu Y., Li J., Xu M., Wei Q., Wang Y. (2015). Biocontrol agent Bacillus amyloliquefaciens LJ02 induces systemic resistance against cucurbits powdery mildew. Front. Microbiol..

[36] Alscher R.G., Erturk N., Heath L.S. (2002). Role of superoxide dismutases (SODs) in controlling oxidative stress in plants. J. Exp. Bot..

[37] Li L., Steffens J.C. (2002). Overexpression of polyphenol oxidase in transgenic tomato plants results in enhanced bacterial disease resistance. Planta.

[38] Pitzschke A., Schikora A., Hirt H. (2009). MAPK cascade signalling networks in plant defence. Curr. Opin. Plant Biol..

[39] Lu K., Guo W., Lu J., Yu H., Qu C., Tang Z., Li J., Chai Y., Liang Y. (2015). Genome-wide survey and expression profile analysis of the mitogen-activated protein kinase (MAPK) gene family in Brassica rapa. PLoS ONE.

[40] Beckers G.J., Jaskiewicz M., Liu Y., Underwood W.R., He S.Y., Zhang S., Conrath U. (2009). Mitogen-activated protein kinases 3 and 6 are required for full priming of stress responses in Arabidopsis thaliana. Plant Cell.

[41] Nianiou-Obeidat I., Madesis P., Kissoudis C., Voulgari G., Chronopoulou E., Tsaftaris A., Labrou N.E. (2017). Plant glutathione transferase-mediated stress tolerance: Functions and biotechnological applications. Plant Cell Rep..

[42] Dalton D.A., Boniface C., Turner Z., Lindahl A., Kim H.J., Jelinek L., Govindarajulu M., Finger R.E., Taylor C.G. (2009). Physiological roles of glutathione S-transferases in soybean root nodules. Plant Physiol..

[43] Sharma R., Sahoo A., Devendran R., Jain M. (2014). Over-expression of a rice tau class glutathione s-transferase gene improves tolerance to salinity and oxidative stresses in Arabidopsis. PLoS ONE.

[44] Chappell J. (1995). Biochemistry and molecular biology of the isoprenoid biosynthetic pathway in plants. Annu. Rev. Plant Biol..

[45] Latif Khan A., Ahmed Halo B., Elyassi A., Ali S., Al-Hosni K., Hussain J., Al-Harrasi A., Lee I.-J. (2016). Indole acetic acid and ACC deaminase from endophytic bacteria improves the growth of Solarium lycopersicum. Electron. J. Biotechnol..

[46] Phillips K., Zaidan F., Elizondo O.R., Lowe K.L. (2012). Phenotypic characterization and 16S rDNA identification of culturable non-obligate halophilic bacterial communities from a hypersaline lake, La Sal del Rey, in extreme South Texas (USA). Aquat. Biosyst..

[47] Wu L., Wu H., Chen L., Xie S., Zang H., Borriss R., Gao X. (2014). Bacilysin from Bacillus amyloliquefaciens FZB42 has specific bactericidal activity against harmful algal bloom species. Appl. Environ. Microbiol..

[48] Kumar S., Stecher G., Li M., Knyaz C., Tamura K. (2018). MEGA X: Molecular evolutionary genetics analysis across computing platforms. Mol. Biol. Evol..

[49] Tamura K., Nei M. (1993). Estimation of the number of nucleotide substitutions in the control region of mitochondrial DNA in humans and chimpanzees. Mol. Biol. Evol..

[50] Sarwar A., Brader G., Corretto E., Aleti G., Abaidullah M., Sessitsch A., Hafeez F.Y. (2018). Qualitative analysis of biosurfactants from Bacillus species exhibiting antifungal activity. PLoS ONE.

[51] Ramarathnam R., Bo S., Chen Y., Fernando W.D., Xuewen G., De Kievit T. (2007). Molecular and biochemical detection of fengycin-and bacillomycin D-producing Bacillus spp., antagonistic to fungal pathogens of canola and wheat. Can. J. Microbiol..

[52] Hajji M., Jarraya R., Lassoued I., Masmoudi O., Damak M., Nasri M. (2010). GC/MS and LC/MS analysis, and antioxidant and antimicrobial activities of various solvent extracts from Mirabilis jalapa tubers. Process Biochem..

[53] Gu Q., Yang Y., Yuan Q., Shi G., Wu L., Lou Z., Huo R., Wu H., Borriss R., Gao X. (2017). Bacillomycin D produced by Bacillus amyloliquefaciens is involved in the antagonistic interaction with the plant-pathogenic fungus Fusarium graminearum. Appl. Environ. Microbiol..

[54] Idris H.A., Labuschagne N., Korsten L. (2007). Screening rhizobacteria for biological control of Fusarium root and crown rot of sorghum in Ethiopia. Biol. Control.

[55] Bawa G., Feng L., Li Y., Shang J., Wu X., Chang X., Sun X., Yu L., Liu C., Du J. (2018). Physiological Analysis Reveals the Possible Resistance Mechanisms of Glycine max to Fusarium solani. J. Agric. Sci..

[56] NCBI. https://www.ncbi.nlm.nih.gov..

[57] Livak K.J., Schmittgen T.D. (2001). Analysis of relative gene expression data using real-time quantitative PCR and the 2− ΔΔCT method. Methods.

